# Protein Signature Predicts Response to Neoadjuvant Treatment With Chemotherapy and Bevacizumab in HER2-Negative Breast Cancers

**DOI:** 10.1200/PO.20.00086

**Published:** 2021-01-28

**Authors:** Mads H. Haugen, Ole Christian Lingjærde, Ingrid Hedenfalk, Øystein Garred, Elin Borgen, Niklas Loman, Thomas Hatschek, Anne-Lise Børresen-Dale, Bjørn Naume, Gordon B. Mills, Gunhild M. Mælandsmo, Olav Engebraaten

**Affiliations:** ^1^ Department of Tumor Biology, Institute for Cancer Research, Division of Cancer Medicine, Oslo University Hospital, The Norwegian Radium Hospital, Oslo, Norway; ^2^ Department of Genetics, Institute for Cancer Research, Division of Cancer Medicine, Oslo University Hospital, The Norwegian Radium Hospital, Oslo, Norway; ^3^ Department of Informatics—Biomedical Informatics, University of Oslo, Oslo, Norway; ^4^ K.G. Jebsen-Centre for B Cell Malignancies, Institute for Clinical Medicine, University of Oslo, Oslo, Norway; ^5^ Department of Clinical Sciences, Division of Oncology and Pathology, Lund University, Lund, Sweden; ^6^ Division of Laboratory Medicine—Pathology, Oslo University Hospital, The Norwegian Radium Hospital, Oslo, Norway; ^7^ Department of Hematology, Oncology and Radiation Physics, Skåne University Hospital, Skåne, Sweden; ^8^ Department of Oncology-Pathology, Karolinska University Hospital, Stockholm, Sweden; ^9^ Institute for Clinical Medicine, Faculty of Medicine, University of Oslo, Oslo, Norway; ^10^ Department of Oncology, Division of Cancer Medicine, Oslo University Hospital, Oslo, Norway; ^11^ Department of Cell, Developmental and Cancer Biology, School of Medicine, Oregon Health Science University, Portland, OR; ^12^ Institute for Medical Biology, Faculty of Health Sciences, University of Tromsø, The Arctic University of Norway, Tromsø, Norway

## Abstract

**PURPOSE:**

Antiangiogenic therapy using bevacizumab has proven effective for a number of cancers; however, in breast cancer (BC), there is an unmet need to identify patients who benefit from such treatment.

**PATIENTS AND METHODS:**

In the NeoAva phase II clinical trial, patients (N = 132) with large (≥ 25 mm) human epidermal growth factor receptor 2 (HER2)-negative primary tumors were randomly assigned 1:1 to treatment with neoadjuvant chemotherapy (CTx) alone or in combination with bevacizumab (Bev plus CTx). The ratio of the tumor size after relative to before treatment was calculated into a continuous response scale. Tumor biopsies taken prior to neoadjuvant treatment were analyzed by reverse-phase protein arrays (RPPA) for expression levels of 210 BC-relevant (phospho-) proteins. Lasso regression was used to derive a predictor of tumor shrinkage from the expression of selected proteins prior to treatment.

**RESULTS:**

We identified a nine-protein signature score named vascular endothelial growth factor inhibition response predictor (ViRP) for use in the Bev plus CTx treatment arm able to predict with accuracy pathologic complete response (pCR) (area under the curve [AUC] = 0.85; 95% CI, 0.74 to 0.97) and low residual cancer burden (RCB 0/I) (AUC = 0.80; 95% CI, 0.68 to 0.93). The ViRP score was significantly lower in patients with pCR (*P* < .001) and in patients with low RCB (*P* < .001). The ViRP score was internally validated on mRNA data and the resultant surrogate mRNA ViRP score significantly separated the pCR patients (*P* = .016). Similarly, the mRNA ViRP score was validated (*P* < .001) in an independent phase II clinical trial (PROMIX).

**CONCLUSION:**

Our ViRP score, integrating the expression of nine proteins and validated on mRNA data both internally and in an independent clinical trial, may be used to increase the likelihood of benefit from treatment with bevacizumab combined with chemotherapy in patients with HER2-negative BC.

## INTRODUCTION

Treatment of solid tumors using antiangiogenic therapy has been explored for several decades.^1,2^ Discovery of vascular endothelial growth factor A (VEGF-A) as a major culprit in tumor angiogenesis led to the development of bevacizumab, a recombinant humanized monoclonal antibody targeting VEGF-A.^3^ Addition of bevacizumab to various chemotherapy regimens has proven highly beneficial in patients with several types of advanced solid tumors resulting in a significant improvement in overall survival (OS) and/or progression-free survival (PFS).

**CONTEXT**

**Key Objective**
The use of anti–vascular endothelial growth factor (VEGF) targeted therapies in combination with chemotherapy in breast cancer (BC) is limited because of the lack of a predictive biomarker. In this study, protein expression in pretreatment tumor biopsies was used to develop a predictor for patients with large BCs.
**Knowledge Generated**
A nine-protein response predictor was established by Lasso regression trained by a continuous response evaluation. The predictor identifies with high significance the patients achieving pathologic complete response and having low residual cancer burden (RCB 0/I). The protein signature was validated using corresponding mRNA expression in the same patient cohort as well as in an independent patient cohort.
**Relevance**
Our study supports using this protein signature as a predictive marker for VEGF inhibition in combination with chemotherapy, and represents a novel opportunity for rational use of this therapy in patients with BC.

Despite good response to bevacizumab therapy in many individual patients with breast cancer (BC), randomly selected treatment populations do not demonstrate improved OS.^4,5^ Thus, the clinical use of bevacizumab in BC is limited and currently only approved in Europe. Regardless of this, activity is observed in subsets of patients pointing to the urgent need for novel biomarkers to select subpopulations in which adequate clinical benefit can be achieved.^6,7^ One of the most obvious and biological plausible biomarkers was the plasma VEGF-A level, and although promising in some studies, evidence from the latter MERiDiAN trial did not support its use for identifying patients with benefit from added bevacizumab.^8^ Other biomarkers at various molecular levels have been investigated such as soluble carbonic anhydrase IX,^9^ *BRCA1/2* mutations,^10^ and DNA methylation signatures.^11^ However, tissue protein expression and protein signatures have not previously been explored, although data at the proteomic level in general have proven highly valuable in drug response prediction models.^12^

In this study, we sought to establish protein expression features predicting the response to treatment with bevacizumab in combination with chemotherapy. To accomplish this, we measured protein expression using reverse-phase protein arrays (RPPA) in tumor samples collected from patients with BC prior to neoadjuvant treatment (NAT) with chemotherapy (CTx) or chemotherapy in combination with bevacizumab (Bev plus CTx), and analyzed expression against treatment effect on tumor size and clinical outcomes. We present our protein signature score (VEGF inhibition response predictor [ViRP] score) showing that patients with pathologic complete response (pCR) or low residual cancer burden (RCB) can be identified in the Bev plus CTx treatment arm, and suggests this group should be offered treatment with bevacizumab or a biosimilar combined with chemotherapy in the future.

## PATIENTS AND METHODS

### Patient Cohort

**FIG A1.**
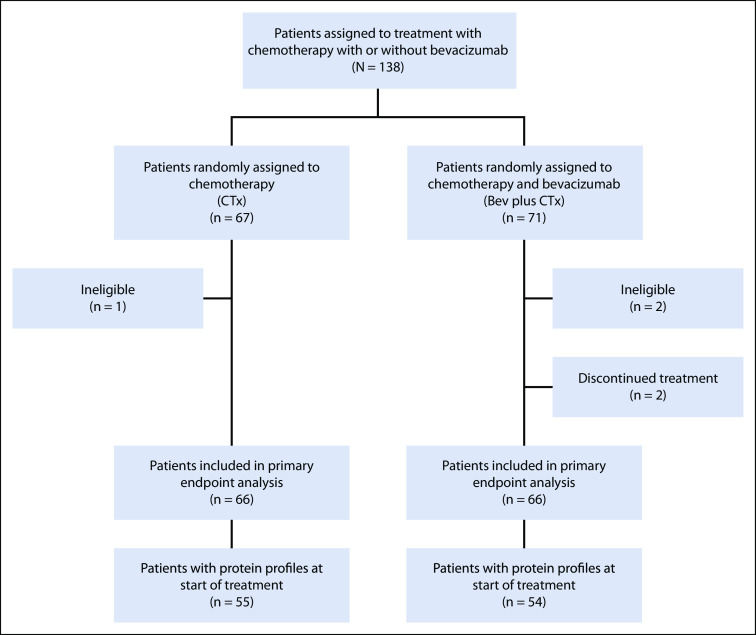
CONSORT diagram.

**FIG A2.**
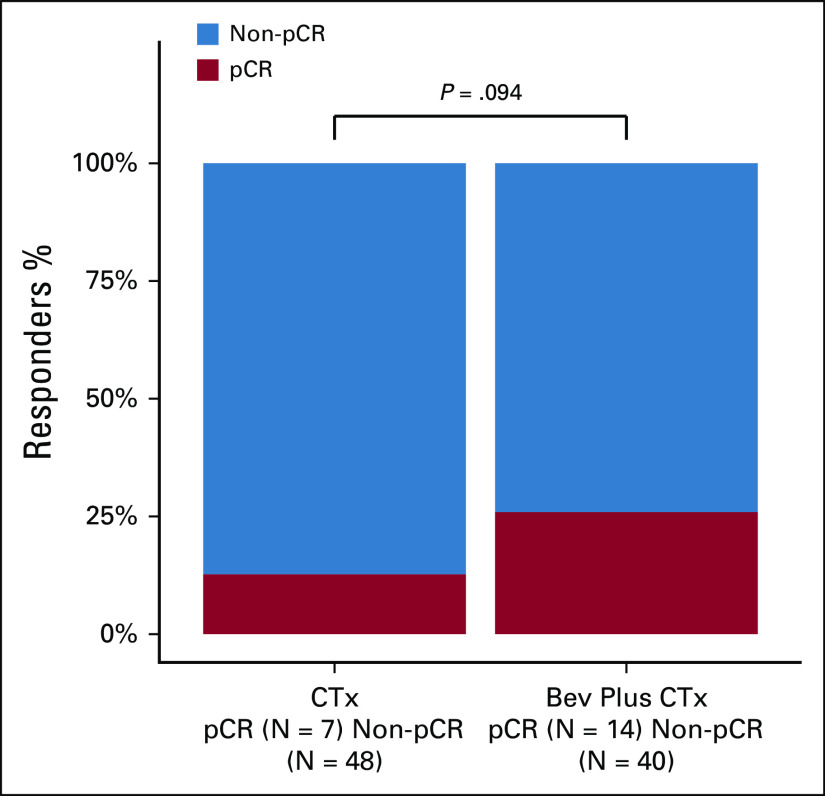
Responders by pCR in study population (N = 109). pCR, pathologic complete response.

To further advance our understanding of response to antiangiogenic NAT, protein expression profiles were established in the NeoAva phase II clinical trial (NeoAva trial, ClinicalTrials.gov identifier: NCT00773695). Patients with human epidermal growth factor receptor 2 (HER2)-negative, previously untreated, breast carcinomas with size ≥ 2.5 cm were included, and 67 and 71 patients randomly assigned to treatment with CTx or Bev plus CTx, respectively. In each treatment arm, 66 patients were included in the primary end point analysis (pCR). One hundred nine samples collected prior to treatment were available for protein analysis on RPPA in the CTx treatment arm (N = 55) and in the Bev plus CTx treatment arm (N = 54) (Appendix Fig A1). Clinicopathologic characteristics of patients, including adverse events, have previously been described.^13^ In brief, the primary end point of the clinical study, pCR, was defined as pathologic stage ypT0 and ypN0 after end of therapy. In the subset of patients with available protein profiles (N = 109), pCR rates were overall higher in the Bev plus CTx (26 %) compared with the CTx (13 %) treatment arm, although not significantly (*P* = .094) (Appendix Fig A2). In addition to pCR, two other response parameters were used in this study. Relative tumor size in percent after 24 weeks of NAT was calculated as tumor size at the time of surgery (longest diameter on histopathologic specimen) relative to tumor size at week 0 (MRI if available or ultrasound or mammography). RCB was calculated using the RCB Calculator,^14^ and dichotomized to low and high for RCB 0/I and RCB II/III, respectively. Tumor cell content in samples was assessed by use of ASCAT^15^ as previously reported.^16^ The study was approved by the Institutional Protocol Review Board, the Regional Ethics Committee, the Norwegian Medicines Agency, performed in accordance with the Declaration of Helsinki, and informed consent was obtained. We further used an external cohort with similar treatment characteristics (PROMIX trial, ClinicalTrials.gov identifier: NCT00957125), with mRNA expression data available,^17^ for validation of the ViRP score.

### Reverse Phase Protein Arrays (RPPA)

Profiling of 210 cancer-relevant proteins of which 54 were in a phosphorylated state (Appendix Table A1) were performed by the RPPA^18^ core facility at MD Anderson Cancer Center (Houston, TX). Tumor protein lysates were serially diluted two-fold for 5 dilutions (from undiluted to 1:16 dilution), probed with antibodies, and visualized by DAB colorimetric reaction. Relative protein levels for each sample were determined and all the data points were normalized for protein loading. All the values were log2 transformed and median centered across each antibody.

### Statistical Analysis and Signature Development

Statistical analyses were conducted using R (v 3.6.3) programming language (R Foundation for Statistical Computing, Vienna, Austria) with RStudio (v 1.2.1335). For development of the ViRP score, the continuous response parameter relative tumor size was used as outcome, whereas the other clinically used response parameters pCR and RCB were used for evaluation of predictive performance. To assess significance of differences in ViRP scores in subgroups, a two-sample *t*-test was applied. To assess significance of correlations between continuous variables, a Spearman or Pearson correlation test was applied, as indicated. All statistical tests were two-sided and a *P*-value < .05 was considered significant. The Fisher’s exact test was used to compare response (pCR and RCB) between groups.

**FIG A3.**
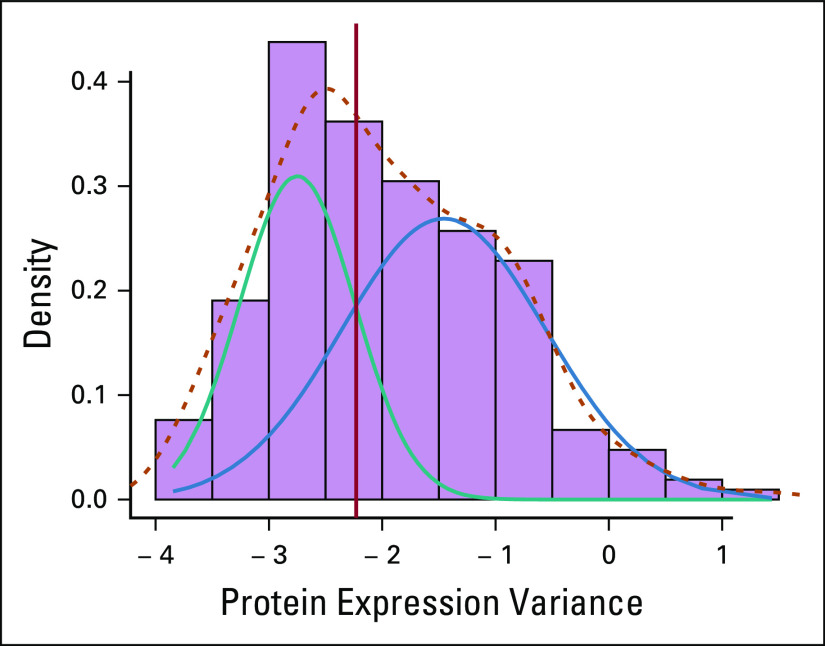
Mixed distribution of protein variance. Mixed distribution of protein expression variances (solid green and blue lines); only proteins with variance above intersection (red line) were selected for input in Lasso regression to determine the ViRP (Appendix Table A3). ViRP, VEGF inhibition response predictor.

Low variance proteins were filtered out by fitting a mixed-model distribution to the protein variances (Appendix Fig A3) using the R-package mixtools.^19^ Adaptive Lasso regression^20^ was performed using the R-package glmnet^21^ in which the penalty parameter lambda was determined by cv.glmnet using the lambda.min value after leave-one-out cross-validation with mean absolute error loss. Receiver operating characteristic (ROC) curves were analyzed using the R-package pROC,^22^ and optimal cutoff was selected based on pCR. Relative importance of each model variable was assessed using the R-package relaimpo^23^ with metrics lmg.

ViRP scores based on mRNA data in the NeoAva and PROMIX study were computed using the intercept and beta-coefficients determined from the protein data in the NeoAva study. The corresponding surrogate mRNA scores were determined using quantile-normalized and probe-averaged mRNA expressions from the genes corresponding to the proteins in the original protein signature, including the phosphoproteins.

## RESULTS

### Protein Profile of Tumors Before Treatment

**FIG A4.**
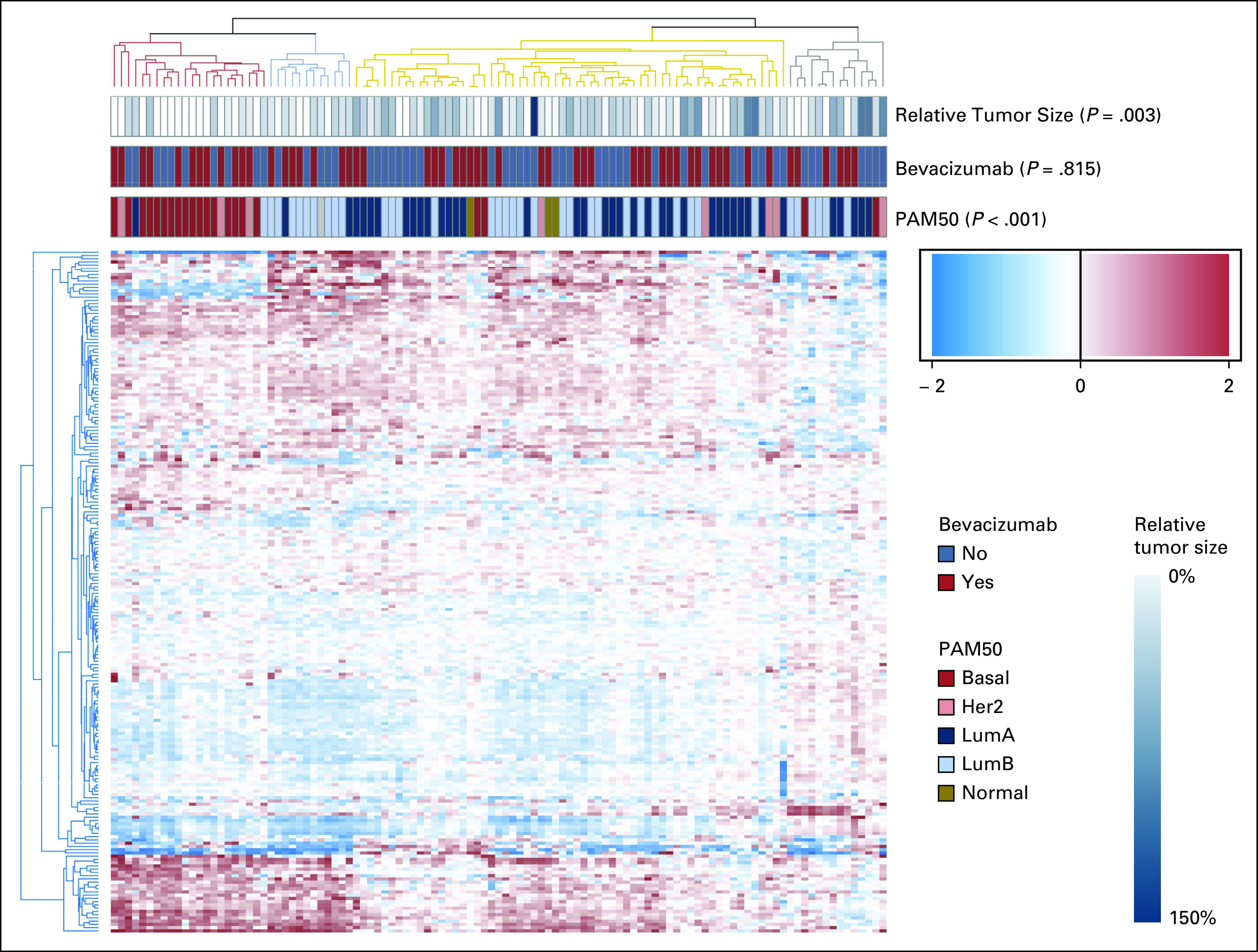
Cluster heatmap of protein expression prior to treatment. Hierarchical clustering (Euclidean distance and complete linkage) of protein expression (red = high to blue = low). Top bars recapitulate relative tumor size, addition of bevacizumab to CTx, and PAM50 classification of tumors. Hierarchical clustering was performed using the R-package Clustermap (https://github.com/cbsteen/clustermap) with median centered and log^2^-transformed RPPA data, and visualized using values normalized to a selected range [− x_0_, x_0_] by application of the transformation f(x) = x_0_tanh(x/x_0_). The number of clusters were determined using the Partitioning Algorithm by Recursive Thresholding (PART) with N = 10,000 permutations (Nilsen et al, Statistical Applications in Genetics and Molecular Biology, 2013).

**FIG A5.**
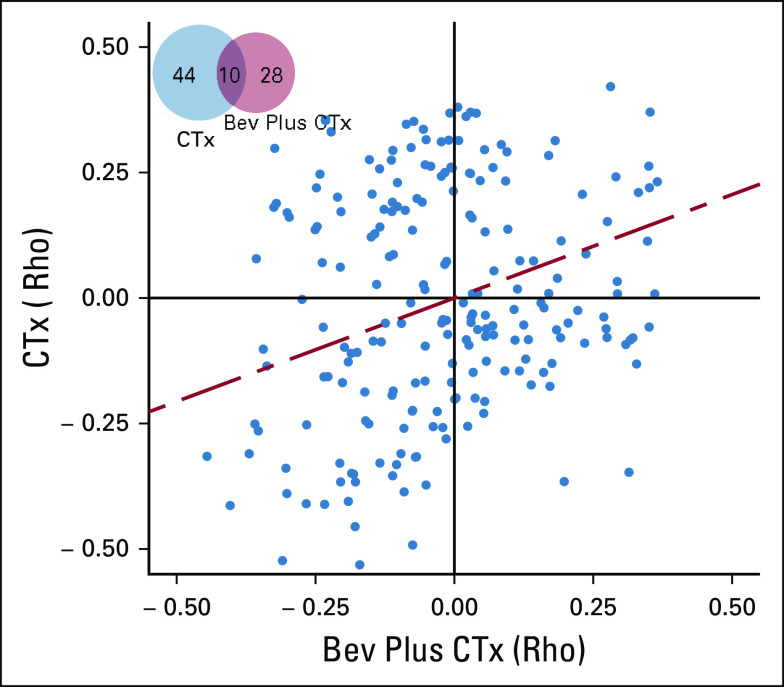
Correlation of protein expression and relative tumor size after treatment. Rho-values derived from the Spearman correlation of protein expression to relative tumor size for patients receiving the Bev plus CTx combination (x-axis) and CTx treatment only (y-axis) with first principal component of relation between the treatment arms (stitched red line). Venn diagram (upper left corner) of proteins in each treatment arm significantly associated with relative tumor size.

Using unsupervised hierarchical clustering on the expression of the 210 proteins in all 109 tumors collected prior to treatment, they cluster into groups significantly related to tumor shrinkage and to PAM50 subtypes, with most estrogen receptor (ER)-negative patients (17 of 21) localizing in a separate subcluster (Appendix Fig A4). Univariate Spearman correlation analysis of protein expressions related to relative tumor size visualized by the non–variable-dependent first principal component demonstrated a positive yet limited relationship between the two treatment arms, with expression of almost half of the proteins (N *=* 103) being inversely correlated with response. The number of proteins significantly (*P* < .05 unadjusted) related to response was higher in the CTx treatment arm (N *=* 54) than in the Bev plus CTx arm (N *=* 38), with only ten in common and of which two had rho-values with opposite signs (Appendix Fig A5 and Appendix Table A2).

### Development of a Protein Signature Score Predicting Response to Treatment With Bev Plus CTx

Addition of bevacizumab to standard treatment with chemotherapy demonstrated benefit compared with chemotherapy alone, although not significant (Appendix Fig A2). To develop a signature predicting response to treatment with Bev plus CTx, protein expression in biopsies taken prior to treatment were used from patients with available relative tumor size after NAT (N *=* 54). Low-variance proteins are likely to have low predictive value, thus a certain level of variance in expression should be present in order for clinical markers to be applicable. We thus reduced the original panel of 210 proteins by plotting the variance in expression and demonstrated a mixed distribution (Appendix Fig A3) where only members belonging mainly to the proteins with higher variance (N *=* 114; Appendix Table A3) were considered for use in the adaptive Lasso regression model. By applying Lasso regression, coupling protein expression to *relative tumor size* (post-NAT), a set of ten proteins with nonzero beta coefficients were discovered. A second iterative round of Lasso on this subset of ten proteins gave the final intercept and beta coefficients, of which one was shrunk to zero giving a final signature of nine proteins with corresponding beta coefficients. Univariate Spearman analysis of the nine proteins demonstrated that with the exception of ACC-pS79, the remaining eight proteins in the ViRP signature were all significantly associated with relative tumor shrinkage (Table 1).

**TABLE 1.**
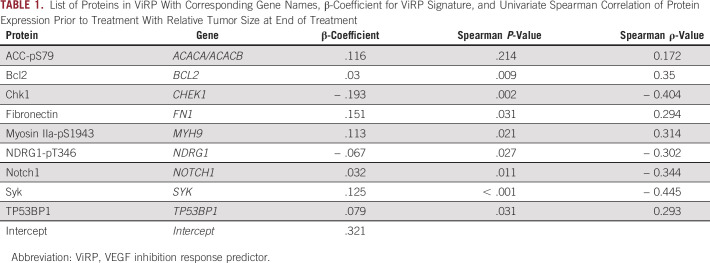
List of Proteins in ViRP With Corresponding Gene Names, β-Coefficient for ViRP Signature, and Univariate Spearman Correlation of Protein Expression Prior to Treatment With Relative Tumor Size at End of Treatment

**FIG 1.**
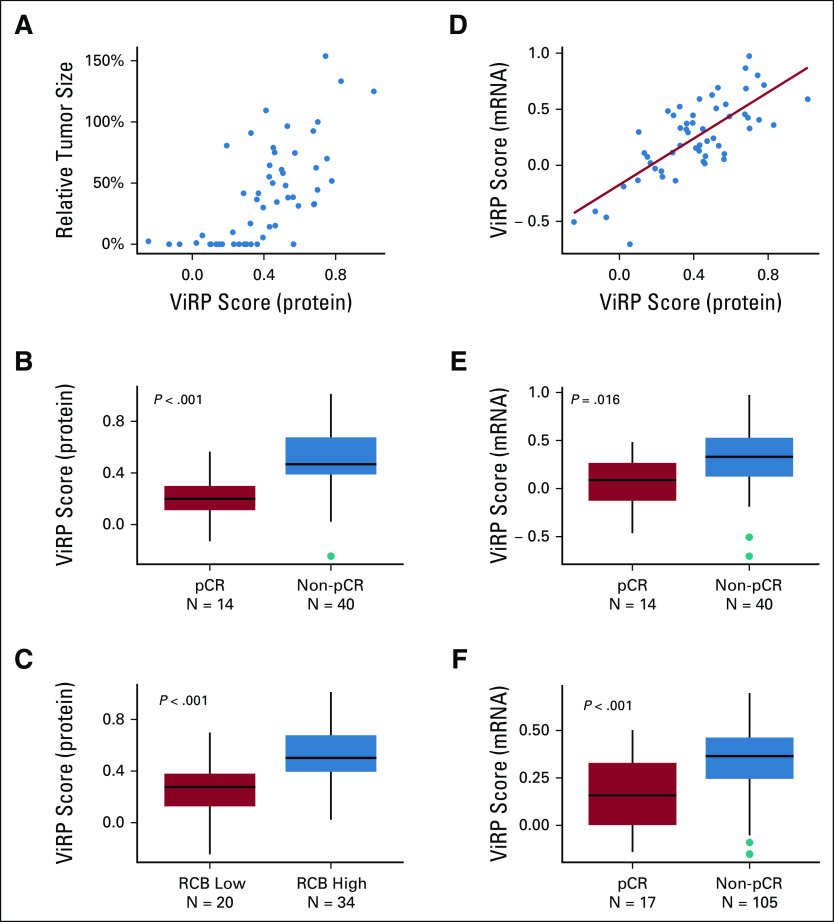
ViRP score based on protein or mRNA in relation to response. (A) ViRP score for each patient with corresponding relative tumor size after end of treatment. (B) ViRP scores in pCR and non-pCR patients. (C) ViRP scores in RCB low (0 and I) and high (II and III) patients. (D) Correlation between protein and mRNA ViRP scores. (E) mRNA ViRP scores in pCR and non-pCR patients in the NeoAva cohort. (F) mRNA ViRP scores in pCR and non-pCR patients in the PROMIX cohort. pCR, pathologic complete response; RCB, residual cancer burden; ViRP, VEGF inhibition response predictor.

The ViRP score for each patient was calculated as the sum of the intercept and beta coefficient weighted expression of the nine proteins. The ViRP score demonstrated significant and high correlation (Pearson R^2^ = 0.67, *P* < .001) with relative tumor size after treatment (Fig 1A). Using pCR as response criteria, the ViRP scores were significantly (*P* < .001) lower in the responding compared to nonresponding patients (Fig 1B). We further evaluated the ViRP score using RCB class as response criteria, as this has been suggested to provide additional and independent prognostic information to yp stage^24^ and has been associated to long-term prognostic risk after neoadjuvant chemotherapy.^25^ Correlation between the ViRP scores and the continuous RCB scores was significant (Pearson R^2^ = 0.39 *P* = .003), and patients having low RCB (class 0 or I) had significantly (*P* < .001) lower ViRP scores than patients with high RCB (class II or III) (Fig 1C). In univariate analysis, only the ViRP score, both as a continuous value and dichotomized (low *v* high), was significantly associated with pCR (Table 2). No significant association was found for tumor stage, PAM50 subtypes, age, PgR, *Tp53* mutation, and nodal or ER status.

**TABLE 2.**
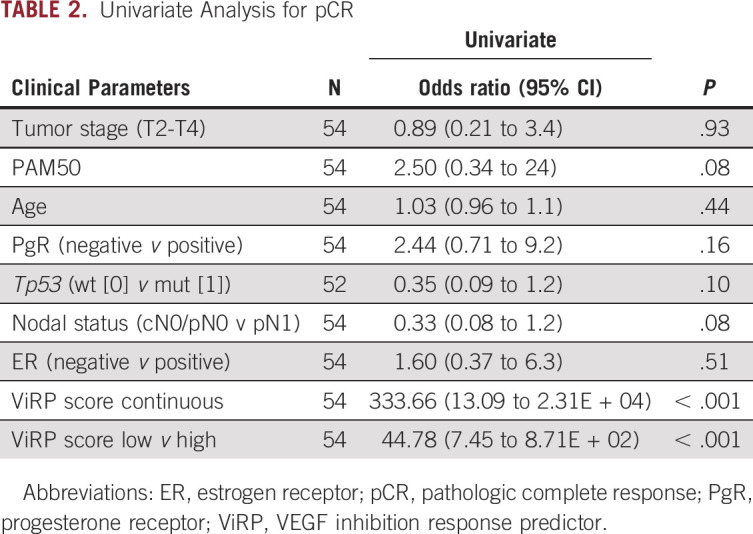
Univariate Analysis for pCR

We next sought to establish and evaluate the corresponding mRNA expression score as a surrogate to the ViRP protein score. By taking the corresponding genes from the ViRP (including the parent protein for the phosphoprotein antibodies) and using the predefined Lasso intercept and beta coefficients, the mRNA score for the NeoAva patients (mRNA ViRP scores) was calculated. Correlation between the mRNA ViRP scores and the original protein ViRP scores in all the 54 evaluated patients was highly significant (Pearson R^2^ = 0.75 *P* < .001) (Fig 1D), and the mRNA ViRP score related to pCR also demonstrated a significantly (*P* = .016) lower score in the pCR compared to non-pCR patients (Fig 1E).

Having demonstrated the use of mRNA as a reasonable surrogate, we sought to validate the protein signature in an external data set obtained from patients in the comparable clinical trial PROMIX (N *=* 122). By calculating the mRNA ViRP score in PROMIX patients and relating it to pCR response, we confirmed significantly (*P* < .001) lower mRNA ViRP scores in the pCR versus non-pCR group (Fig 1F).

### Predictive Performance of ViRP Scores

**FIG 2.**
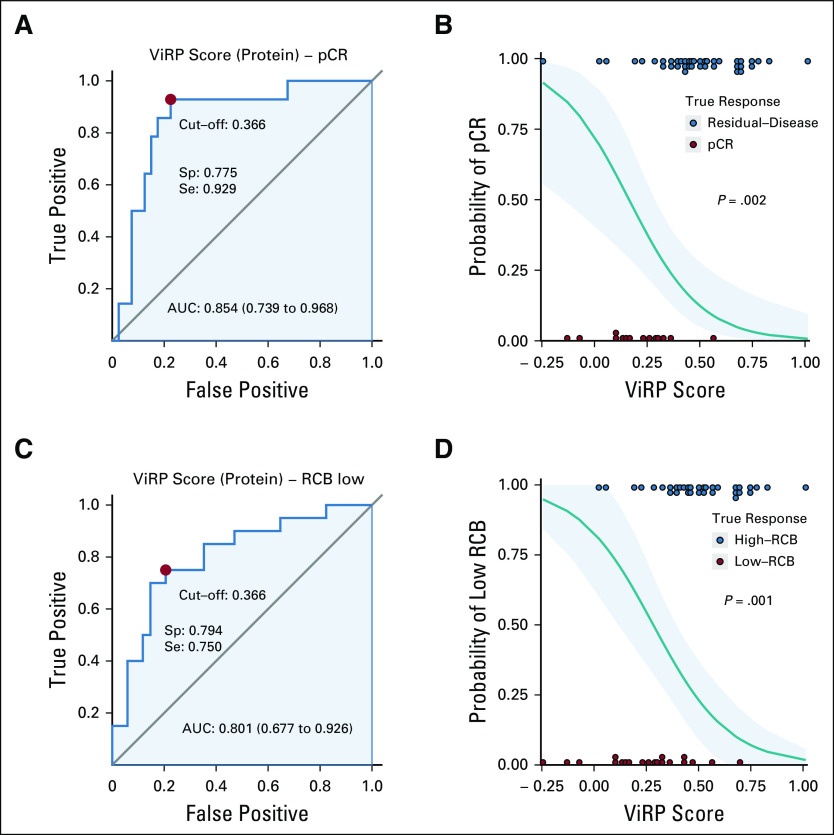
Predictive performance of the ViRP score. (A) ROC curve for ViRP score prediction of pCR. (B) ViRP score prediction of probability for pCR (green line) with 95% CI (blue contour). True response by pCR (red dots) marked on bottom and non-pCR (blue dots) marked on top. (C) ROC curve for ViRP score prediction of low RCB (0 and I). (D) ViRP score prediction of probability for low RCB (0 and I) (green line) with 95% CI (blue contour). True response by low RCB (red dots) marked on bottom and high RCB (blue dots) marked on top. AUC, area under the curve; pCR, pathologic complete response; RCB, residual cancer burden; ROC, receiver operating characteristic; ViRP, VEGF inhibition response predictor.

**FIG A6.**
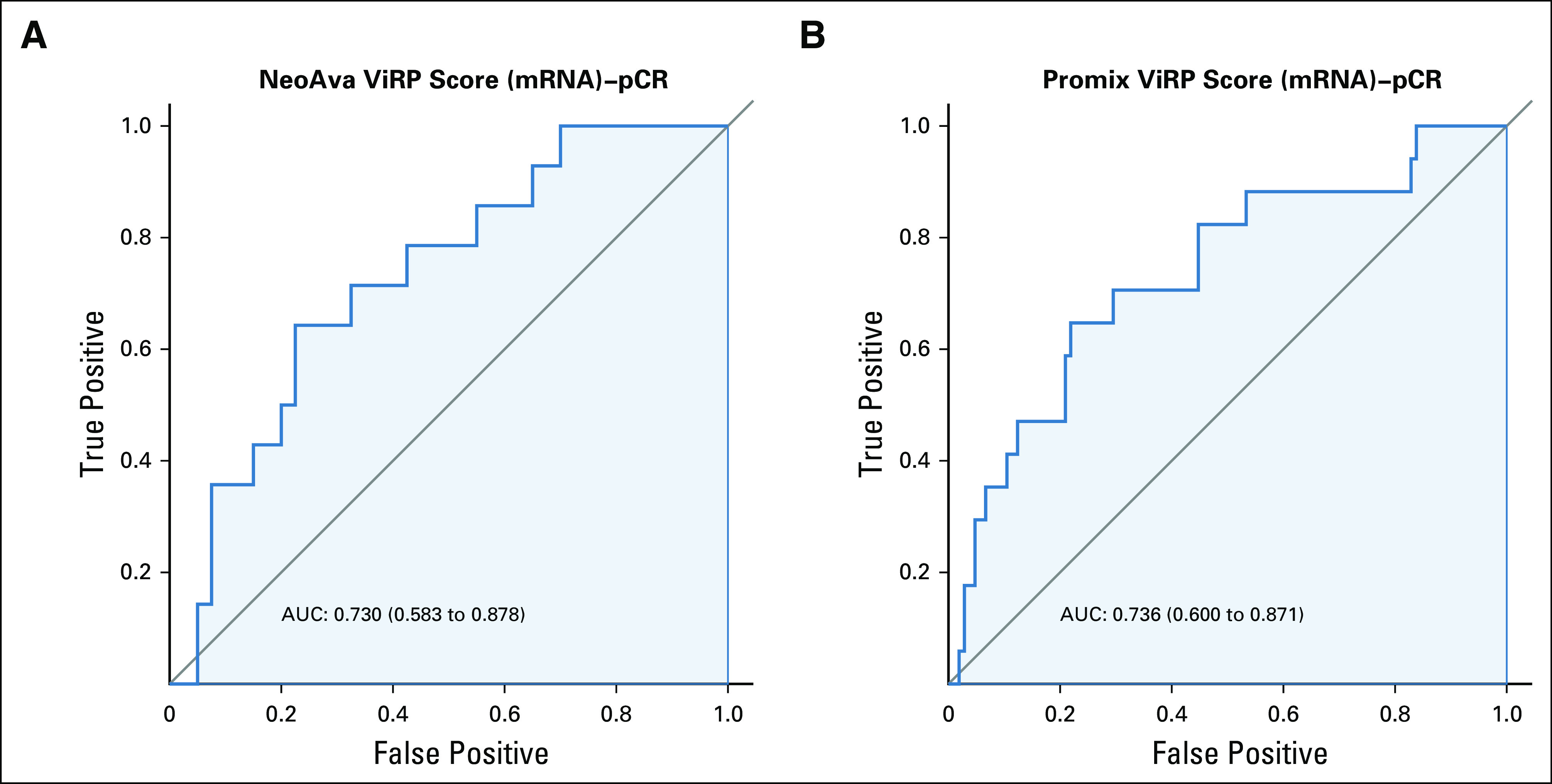
ROC curves for prediction of pCR with mRNA ViRP scores in the NeoAva and PROMIX trials. (A) ROC curve for mRNA ViRP score prediction of pCR in NeoAva. (B) ROC curve mRNA ViRP score prediction of pCR in PROMIX. AUC, area under the curve; pCR, pathologic complete response; ROC, receiver operating characteristic; ViRP, VEGF inhibition response predictor.

The predictive performance of the ViRP score was evaluated using ROC curves. The predictive accuracy (area under the curve [AUC]) of the ViRP score for pCR and low RCB was 0.85 (CI, 0.74 to 0.97) and 0.80 (CI, 0.68 to 0.93), respectively (Fig 2A and C). Similar results were obtained assessing the mRNA ViRP scores in NeoAva and PROMIX, demonstrating AUCs of 0.73 (CI, 0.58 to 0.88) and 0.74 (CI, 0.60 to 0.87), respectively (Appendix Fig A6). Additionally, using binomial modeling in a reverse approach, the ViRP score was evaluated for its ability to predict the probability of pCR or low RCB (Fig 2B and D), which demonstrated significant results (*P* = .002 and *P* = .001, respectively).

**FIG 3.**
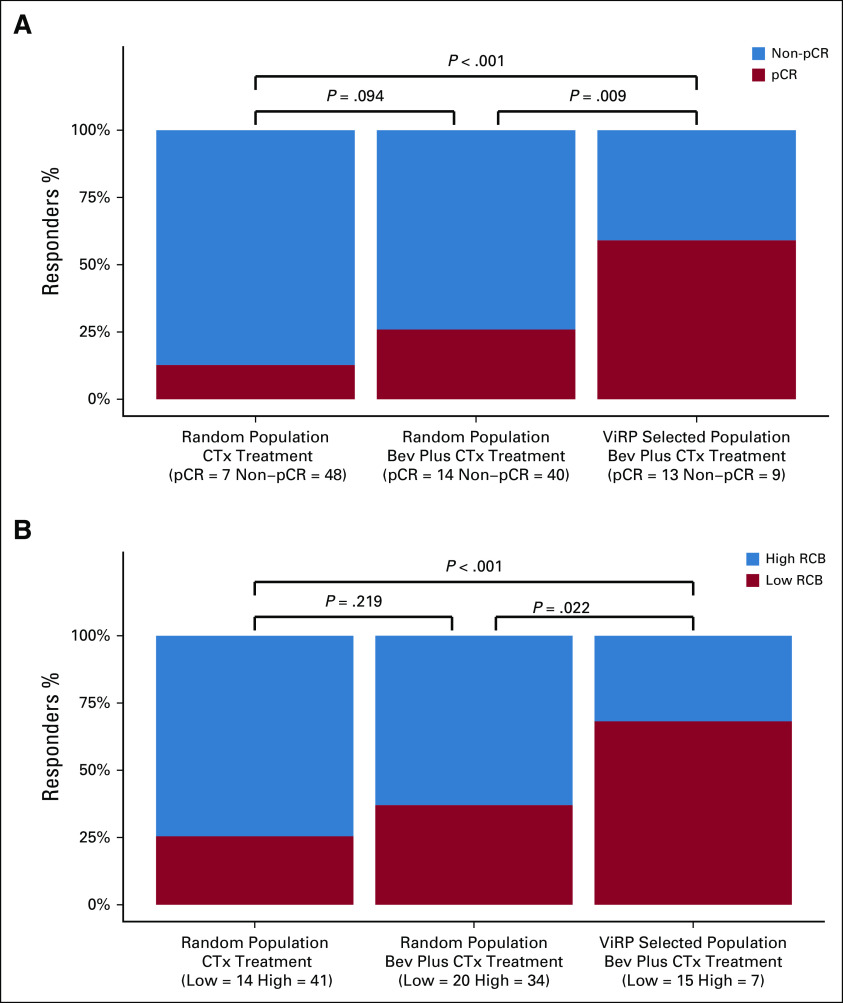
Response rates by selection of patients using the ViRP score. (A) Fraction of patients obtaining pCR when treated with standard CTx in all NeoAva patients (N = 55) or Bev plus CTx in all NeoAva patients (N = 54) and selected based on ViRP score (N = 22). (B) Fraction of patients obtaining low RCB (0 and I) when treated with standard CTx in all NeoAva patients (N = 55) or Bev plus CTx in all NeoAva patients (N = 54) and selected based on ViRP score (N = 22). pCR, pathologic complete response; RCB, residual cancer burden; ViRP, VEGF inhibition response predictor.

To assess the potential clinical benefit of using the ViRP score to select patients for treatment with Bev plus CTx, the score value determined by ROC-analysis for optimal balance between true and false pCR was used as the cutoff (0.366). The fraction of patients responding by pCR or low RCB was compared in all patients versus the ViRP score selected patient population. This demonstrated a significant increase, approximately doubling, in the percentage of both pCR responders (*P* = .009) and low RCB (*P* = .022) in the ViRP score selected patient population. Use of the ViRP score for selecting patients to Bev plus CTx demonstrated a clear benefit with an increased response both by pCR (*P* < .001) and RCB (*P* < .001) compared with standard CTx treatment (Fig 3).

## DISCUSSION

We have defined a protein signature score named ViRP for use in large HER2-negative primary BCs that identifies good responders to NAT with chemotherapy plus bevacizumab. Use of the ViRP score for selection to Bev plus CTx therapy significantly enriches for patients responding to treatment (pCR or RCB-low). Importantly, the robustness of the ViRP score was confirmed through validation in an independent clinical cohort (PROMIX).

**FIG A7.**
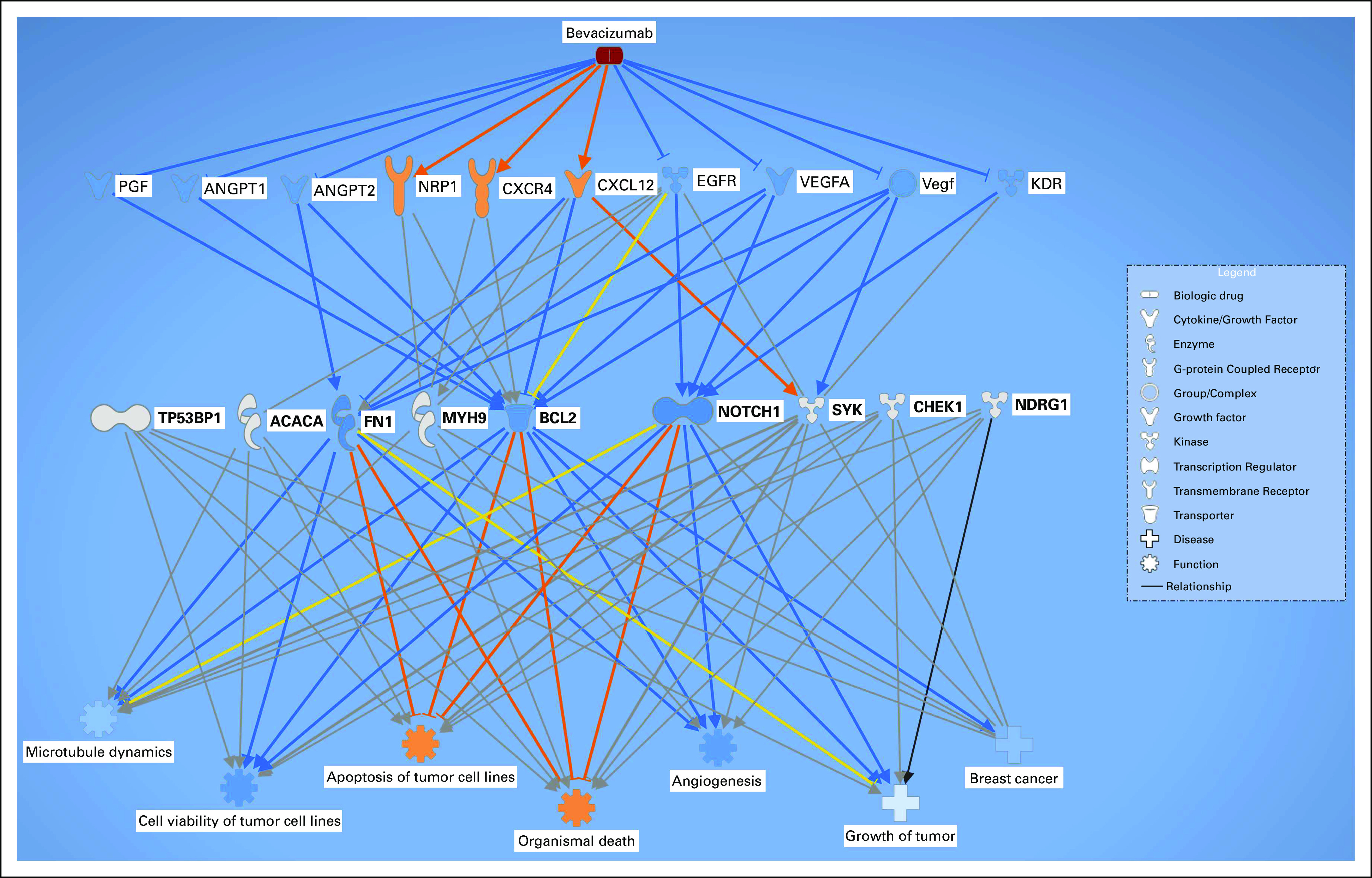
In silico predicted downstream biological effects of bevacizumab treatment mediated through the ViRP proteins. In silico simulation with Ingenuity Pathway Analysis (version 47547484, Qiagen) was used to predict biologic effects (lower row) affected by the ViRP member proteins (second bottom row) when influenced by bevacizumab treatment (top row) through a set of intermediate proteins (second top row). Red or orange color predicts activation, blue color indicates inhibition, and yellow color indicates findings inconsistent with the state of downstream molecule. ViRP, VEGF inhibition response predictor.

The Lasso regression method was used to select proteins for the ViRP score. Like other variable selection methods, Lasso determines variables based on inferred association strength. Further studies would be required to establish with certainty a causal relationship between the selected proteins and patient outcome. However, in silico evaluation of the ViRP proteins under stimulus of bevacizumab treatment links them to response on relevant biological processes (Appendix Fig A7).

**FIG A8.**
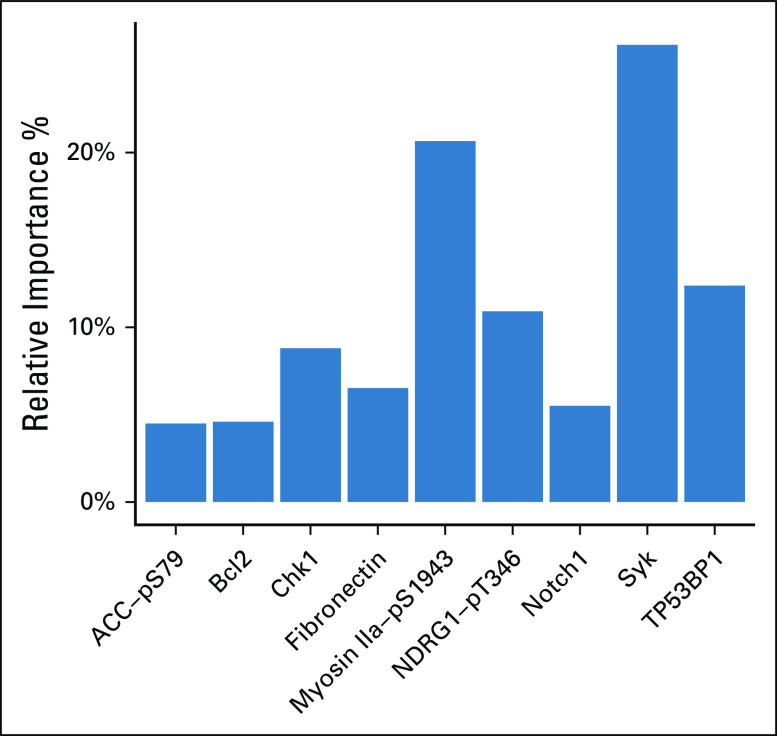
Relative importance of proteins in ViRP signature. Relative contribution of each protein in the ViRP signature for prediction of pCR. pCR, pathologic complete response; ViRP, VEGF inhibition response predictor.

Ranking the relative importance of each protein in the ViRP signature is interesting with respect to, for example, the potential to use each or selected proteins as biomarkers separately. It appears that the Syk protein is of high importance (Appendix Fig A8), which also coincides with being the only protein in the signature differently expressed (*P* = .002) in pCR versus non-pCR patients. The Syk protein is enriched in immune cells, but also expressed in breast epithelium and endothelial cells where it plays a role in angiogenesis.^26^

**FIG A9.**
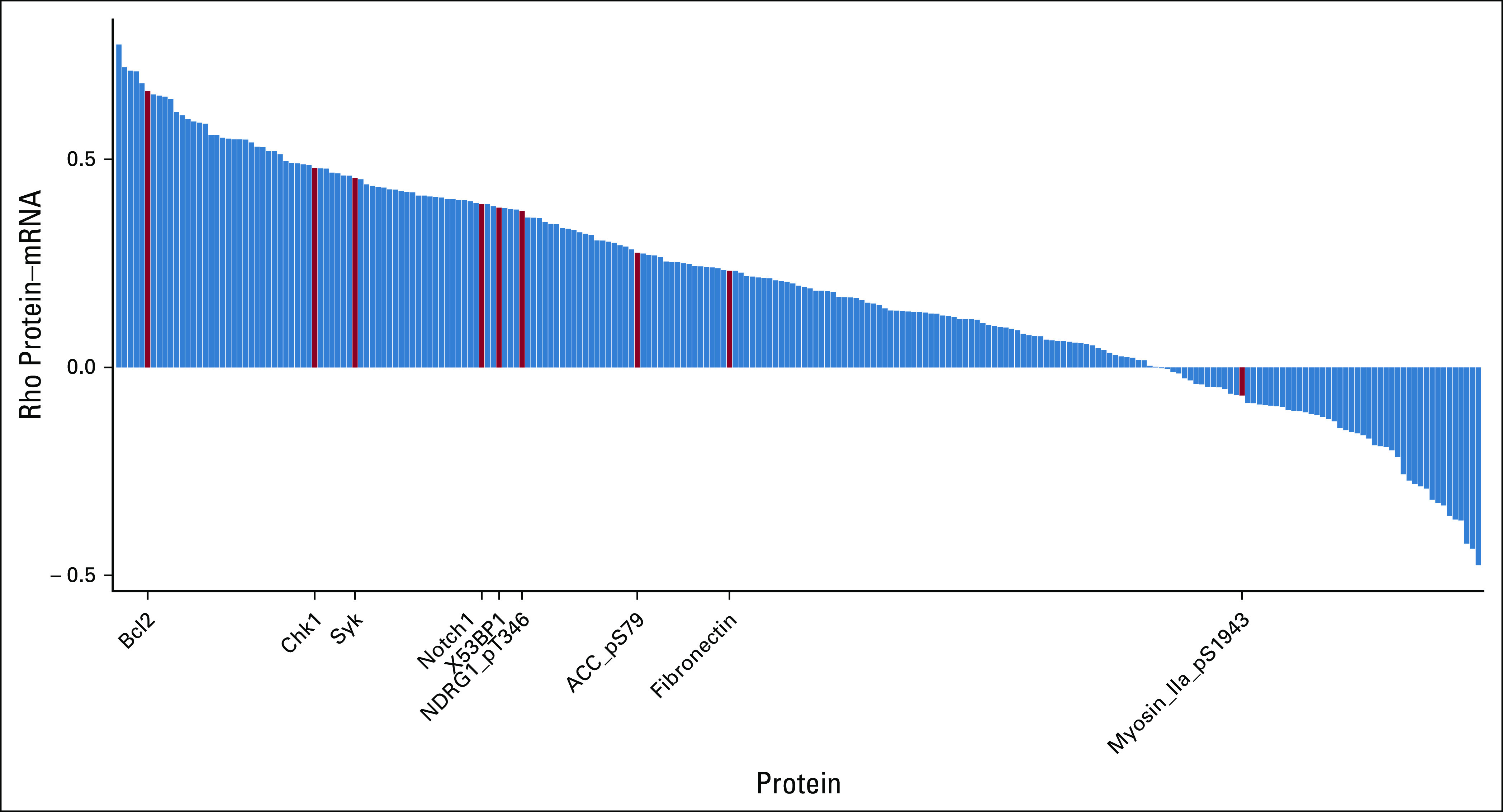
Correlation between protein and mRNA expression. Rho-Spearman correlation of all individual protein-mRNA in the data set (N = 210) with ViRP proteins highlighted in red. ViRP, VEGF inhibition response predictor.

When passing from protein to mRNA expression, the functional dimension of activating or deactivating phosphorylations is lost. This potentially influences the performance of the mRNA ViRP score when used as surrogate for the original protein score, and could partly explain the observed drop in AUC for the ROC analysis between the protein and mRNA data sets in the NeoAva trial (Fig 2A and Appendix Fig A6A). Spearman correlation between expression of all 210 assessed proteins and mRNAs showed that of the nine proteins in the signature, the four with the lowest correlation were phosphorylated or extracellularly localized proteins. However, the signature members had a significantly (*P* = .039) higher correlation compared with all proteins in the original data set (Appendix Fig A9). Although the NeoAva protein data set is too small to draw conclusions, a previous observation of nearly 10,000 proteins in BC showed that established prognostic mRNA-centered signatures (ie, PAM50-ROR, Oncotype DX, and MammaPrint) in general had higher protein-mRNA correlation than average for the human genome.^27^ We propose that because of the closer relation proteins have to phenotype, biomarker signatures being developed to identify features with clinical, and thus phenotypical impact, will represent genes with high expressional and translational penetrance.

In the NeoAva clinical trial, patient subgroups were divided based on ER expression (using 1% positivity as cutoff), and better response (as assessed by pCR and RCB) was in general observed for the ER-negative group, whereas only the ER-positive group had significant benefit of added bevacizumab. Furthermore, in univariate analysis, ER protein expression was significantly correlated with relative tumor size after treatment in the Bev plus CTx-treated patients. Nevertheless, the absence of ER expression being selected for the protein signature indicates that ER may not be of major importance for the response to treatment with Bev plus CTx. This is also consistent with the discrepancy seen in large studies regarding the benefit of added bevacizumab in the ER-positive or ER-negative subpopulations of patients.^28-30^

**FIG A10.**
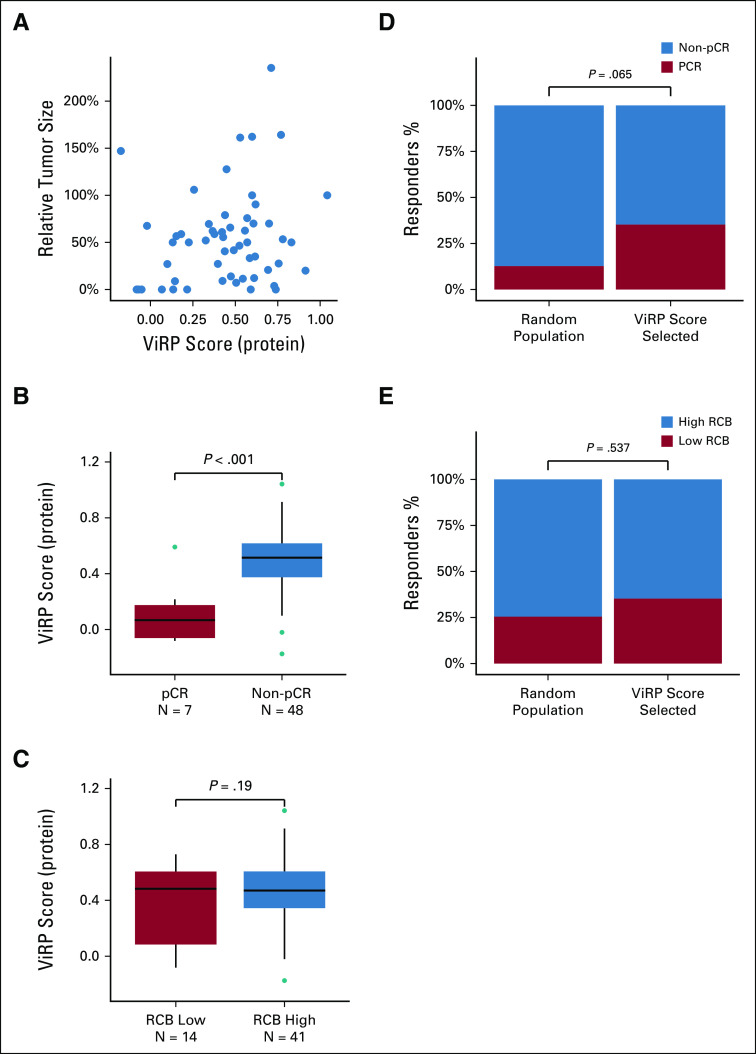
ViRP scores in the CTx treatment arm. (A) ViRP score for each patient with corresponding relative tumor size. (B) ViRP scores in pCR and non-pCR patients. (C) ViRP scores in RCB low (0 and I) and high (II and III) patients. (D) Fraction of patients obtaining pCR when treated with standard CTx in all NeoAva patients (N = 55) or selected based on ViRP score (N = 17). (E) Fraction of patients obtaining low RCB (0 and I) when treated with standard CTx in all NeoAva patients (N = 55) or selected based on ViRP score (N = 17). pCR, pathologic complete response; RCB, residual cancer burden; ViRP, VEGF inhibition response predictor.

The relative higher number of proteins significantly correlated with tumor size after treatment in the CTx arm (N *=* 54) suggested that a similar protein signature predicting response to chemotherapy alone might be developed. However, use of adaptive Lasso regression only returned a signature of two proteins with low predictive performance, and not represented in the Bev plus CTx ViRP signature. Use of the ViRP score developed for Bev plus CTx treatment applied on the CTx patients did demonstrate a significantly lower score in the limited number of patients with pCR (N *=* 7), but not when assessed for the low-RCB patients (N *=* 14). Use of the ViRP score for selecting patients to CTx treatment did not significantly enrich for pCR or RCB responders (Appendix Fig A10). This does not exclude the possibility that the proteins in the ViRP signature also reflect sensitivity to the baseline chemotherapy when combined with bevacizumab.

**FIG A11.**
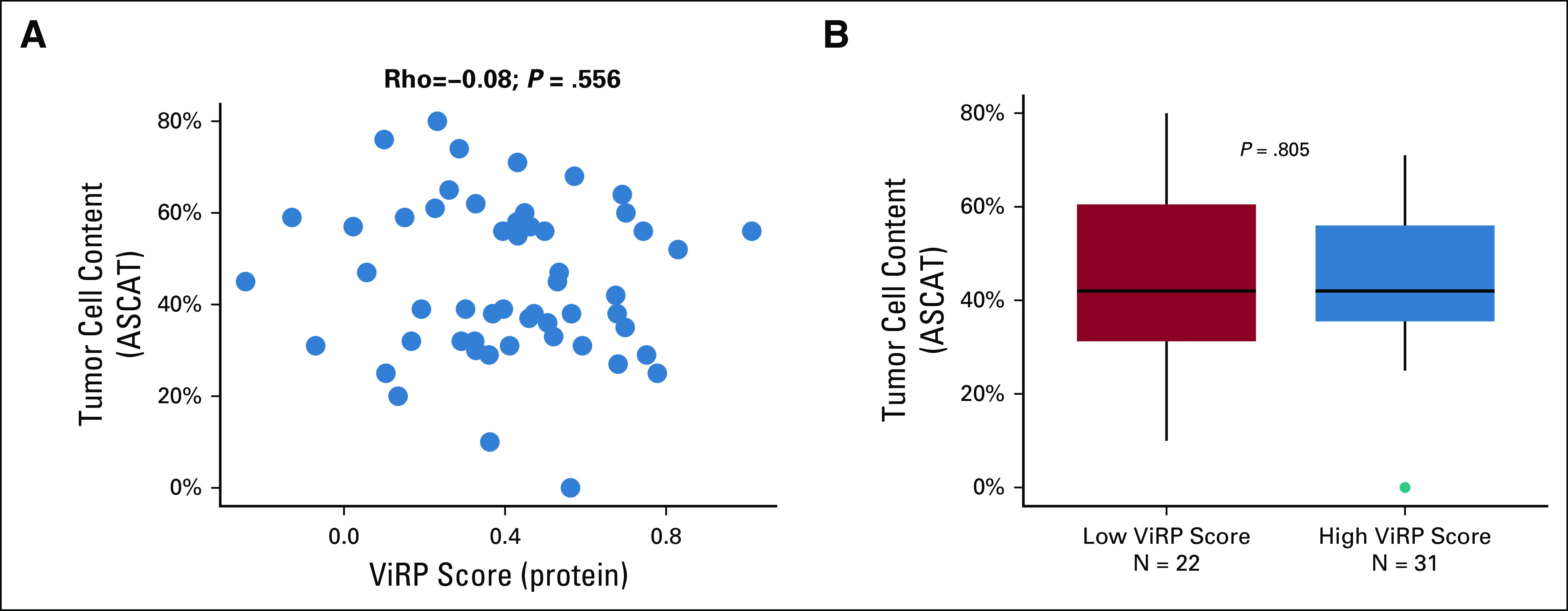
ViRP score in relation to tumor cell content. (A) ViRP score for each patient with corresponding tumor cell content. (B) Tumor cell content in patients with low or high ViRP score. ViRP, VEGF inhibition response predictor.

Bulk tumor samples may have variable tumor cell content, which might influence the readout of mRNA or protein-based molecular signatures.^31^ Influence of tumor cell content (median of 42%) on the ViRP signature, or on its individual proteins, did not demonstrate any significant associations in the Bev plus CTx-treated patients (Appendix Fig A11). Thus, the ViRP score is not highly reactive to tumor purity, although a minimum of tumor cells is likely to be required. The current study has not investigated how this affects the predictive value of the ViRP score, and this will be evaluated in ongoing studies. Furthermore, the ViRP score should be related to the mechanism of action of the anti-VEGF therapy, which involves normal tissue constituents and in particular endothelial cells and vessels.^32^ Thus, a composite tissue is likely required for the score to function. Despite the validation in an independent clinical trial, uncertainty about this composite score will remain until proven effective in a new clinical trial.

Use of Bev plus CTx as first-line treatment in patients with locally advanced or metastatic BC (mBC) has in the RIBBON-1 study shown improved tumor response and clinical benefit in terms of PFS.^33^ Furthermore, real-life data in patients with mBC have also indicated a significant effect on OS from treatment with bevacizumab in combination with chemotherapy.^34^ The ViRP score is predicting responses, but further studies are warranted to assess for impact on survival. Although the discovered ViRP signature was established for patients treated with Bev plus CTx in a neoadjuvant setting, it recapitulates biology related to treatment response and could have the potential to also predict response to Bev plus CTx therapy in the metastatic setting. Additionally, studies are initiated to investigate whether the ViRP signature recapitulates common response biology shared by other solid cancer types for which bevacizumab in combination with chemotherapy is commonly used.^35^

## Figures and Tables

**TABLE A1.**
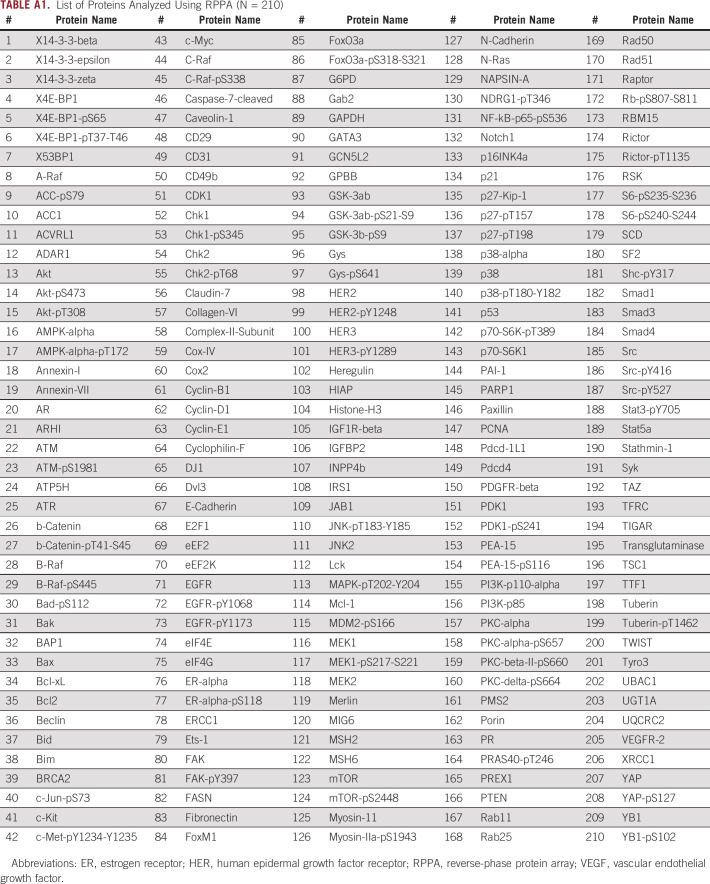
List of Proteins Analyzed Using RPPA (N = 210)

**TABLE A2.**
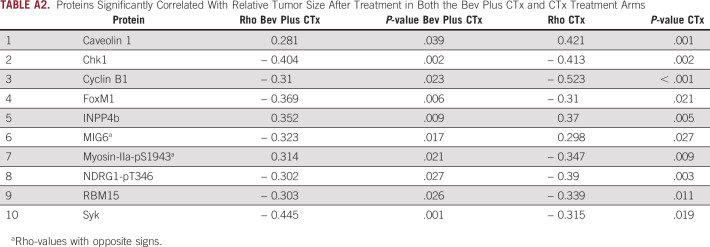
Proteins Significantly Correlated With Relative Tumor Size After Treatment in Both the Bev Plus CTx and CTx Treatment Arms

**TABLE A3.**
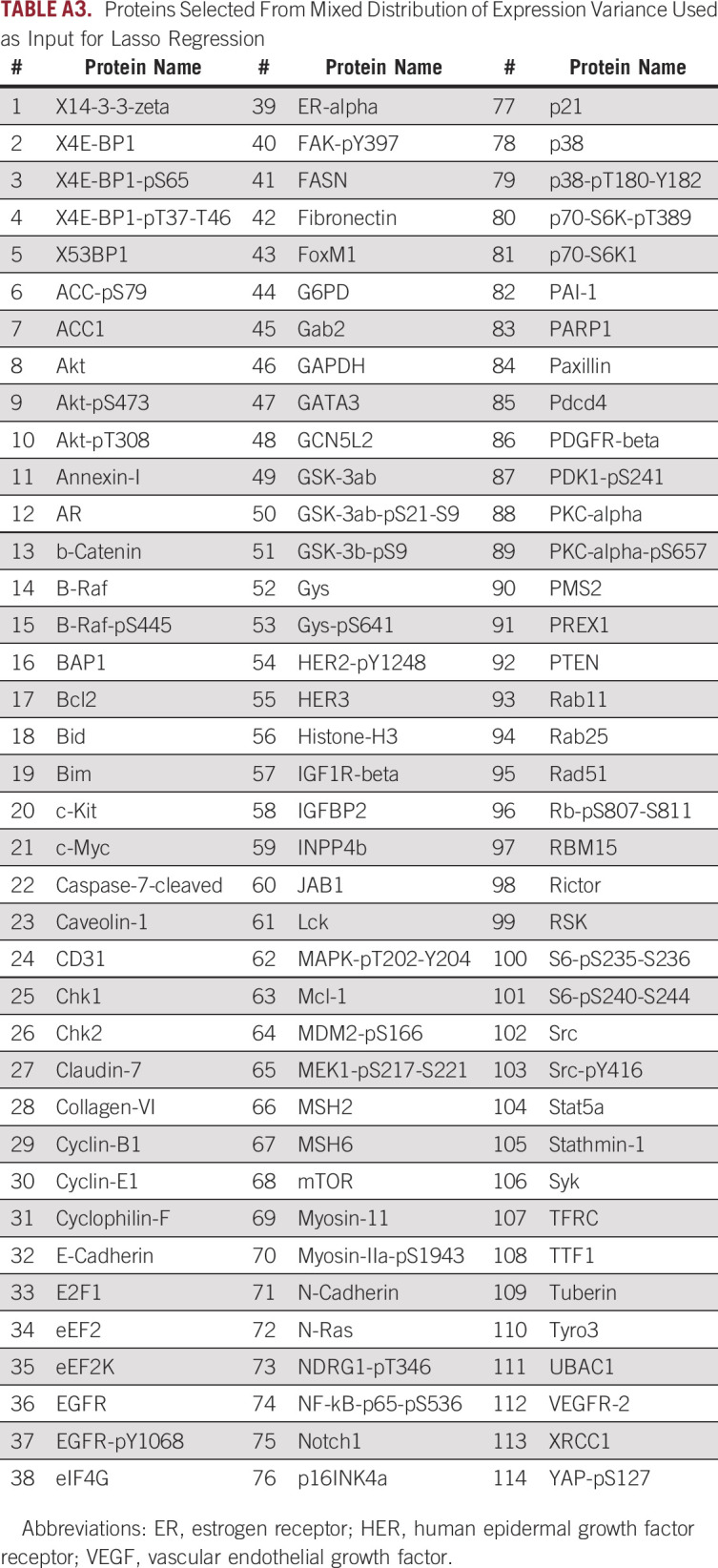
Proteins Selected From Mixed Distribution of Expression Variance Used as Input for Lasso Regression
